# HoxB8 neutrophils replicate Fcγ receptor and integrin‐induced neutrophil signaling and functions

**DOI:** 10.1002/JLB.1AB0618-232R

**Published:** 2018-09-13

**Authors:** Julia Y. Chu, Barry McCormick, Greta Mazelyte, Melina Michael, Sonja Vermeren

**Affiliations:** ^1^ The MRC Centre for Inflammation Research University of Edinburgh Edinburgh United Kingdom

**Keywords:** granulocytes, human cell lines, monocytes/macrophages, neutrophils, adhesion molecules, intracellular signaling, Fc Receptors, transgenic/knockout mice

## Abstract

Neutrophils are short‐lived, terminally differentiated leukocytes that form an essential part of host immunity and play a key role in acute and chronic inflammation. The analysis of these important cells is hindered by the fact that neutrophils are not amenable to culture, transfection, or transduction. Conditionally HoxB8‐immortalized mouse hematopoietic progenitors are suitable for in vitro differentiation of a range of myeloid cells, including neutrophils. Integrins and FcγRs are cell surface receptors, the ligation of which is required for a range of neutrophil functions that are important in health and disease. We show here that HoxB8 neutrophils express major neutrophil integrins and FcγRs. They respond to FcγR and integrin stimulation in a manner that is comparable with primary neutrophils, in terms of intracellular signaling. HoxB8 neutrophils also perform a range of FcγR/integrin‐dependent neutrophil functions, including, generation of reactive oxygen species, degranulation, and chemotaxis. Our findings suggest that HoxB8 neutrophils represent a faithful experimental model system for the analysis of Fc and integrin receptor‐dependent neutrophil functions.

**Abbreviations**
4OHT: 4‐hydroxy‐tamoxifen
BMN: bone marrow‐derived neutrophil
CRISPR: clustered regularly interspaced short palindromic repeats
fMLF: N‐formyl‐methionyl‐leucyl‐phenylalanine
IC: immune complex
pRGD: polyArg‐Gly‐Asp
ROS: reactive oxygen species

## 1 INTRODUCTION

Neutrophils, the most abundant circulating leukocytes in man, are an essential component of the innate immune system.^1^, ^2^ Upon activation neutrophils extravasate, chemotaxing to sites of infection or sterile injury, following gradients of chemoattractants. To fulfil their indispensable function in host defense, neutrophils phagocytose and kill pathogens. They generate reactive oxygen species (ROS) and degranulate, releasing cytotoxic compounds and proteases. Where activated in a poorly controlled manner, neutrophilic inflammation can drive significant host injury. Integrins and Fc receptors are cell surface receptors that are critical for neutrophil functions such as recruitment and phagocytosis.^3^ Immune complexes (ICs) that have precipitated onto biologic surfaces act as important activators of FcγRs in combination with integrins, driving significant neutrophilic inflammation as exemplified by a range of autoimmune diseases such as rheumatoid arthritis.^4^, ^5^

Terminally differentiated neutrophils are constantly renewed in the bone marrow. Neutrophils are programmed to undergo constitutive apoptosis after only a short time in the circulation, with estimated lifespans between 1 and 5 d.^6^, ^7^ Following isolation, neutrophils rapidly undergo apoptosis, with significant cell death observed after only 6–12 h in culture. Although large numbers of primary neutrophils can easily be obtained, this places a distinct limit onto in vitro experimentation. In particular, neutrophils are not amenable to genetic modification. Genetically modified mice represent the perhaps most widely used model system for the analysis of neutrophil functions. In addition to permitting the in vitro characterization of freshly isolated primary neutrophils carrying any genetic modification of interest, mice also offer the advantage of functional in vivo analyses. The generation of new mouse strains is, however, time consuming and expensive.

Conditional immortalization of mouse bone marrow or fetal liver‐derived hematopoietic progenitor cells with a retrovirally expressed HoxB8‐ER fusion construct has been described.^8^ Depending on the cytokines employed, HoxB8‐immortalized progenitors can be used for the in vitro differentiation of a range of leukocytes, notably, HoxB8 neutrophils,^8^, ^9^, ^10^, ^11^ but also macrophages, basophils, and lymphocytes.^9^, ^10^, ^11^, ^12^ Conditionally HoxB8‐immortalized progenitors can be generated from genetically modified mice. The progenitors are moreover amenable to genetic manipulation by viral transduction,^11^, ^13^, ^14^ enabling the generation of genetically modified near primary neutrophils. Once differentiated, HoxB8 neutrophils were shown to exhibit characteristic morphologic and functional features of primary neutrophils. HoxB8 neutrophils are moreover characterized by terminal differentiation, undergoing constitutive apoptosis.^14^, ^15^ Indeed, differentiated HoxB8 neutrophils were reported to recapitulate neutrophil function even in vivo, with differentiated as well as undifferentiated progenitors reported in adoptive transfer experiments.^13^, ^14^ In the context of in vitro analyses performed by a number of different laboratories, HoxB8 neutrophils efficiently phagocytosed yeast and zymosan particles,^13^ released cytokines^14^, ^15^ and generated extracellular traps.^16^ The functionality of HoxB8 neutrophils in integrin and/or FcγR‐mediated adhesion‐dependent processes, however, remains to be explored.

We show here that HoxB8 neutrophils represent a useful model for the analysis of integrin and FcγR‐stimulated neutrophil functions. Hence, HoxB8 neutrophils exhibit similar FcγR and integrin receptor expression as primary neutrophils. HoxB8 neutrophils chemotax, and their FcγR and integrin receptor stimulation induces molecular signaling events as well as production of ROS and degranulation in a manner that resembles primary neutrophils.

## 2 MATERIALS AND METHODS

All reagents were of the highest available grade and with lowest possible endotoxin level. Cell culture reagents were from (Life Technologies, Paisley, UK) cultureware from (Corning, Amsterdam, The Netherlands) and other reagents from (Sigma, Gillingham, UK) unless indicated otherwise.

### 2.1 Cell culture

Cells were cultured at 37°C with 5% CO_2_ in a humidified incubator. HoxB8 conditionally immortalized progenitors were generated from bone marrows of C57Bl/6 mice essentially as described^8^; see supplemental information for a detailed description. HoxB8 progenitors were differentiated in the presence of GM‐CSF, resulting in mixed neutrophils (∼60%) and monocytes/macrophages. Where indicated, HoxB8 neutrophils were passed over a discontinuous percoll gradient (25%, 42%, and 51%^17^) for increased purity (>80%). Prior to experiments cells were washed into assay buffer.

### 2.2 Isolation of primary neutrophils from bone marrow

Mouse bone marrow‐derived neutrophils (BMNs) were prepared from hind legs of C57Bl/6 mice by employing a discontinuous percoll gradient as previously described.^18^ BMNs were of approximately 70% purity according to morphologic analysis of cytocentrifuge preparations. Mouse experiments were approved by the University of Edinburgh animal welfare committee and conducted under control of the UK Home Office (PPL 60/4502 and PFFB42579).

### 2.3 Preparation of ICs and of fibrinogen‐coated plastic

Insoluble ICs (HSA and rabbit polyclonal IgG to HSA) were prepared as mentioned in Chu et al.^19^ Immobilized ICs (BSA and rabbit polyclonal to BSA) and fibrinogen‐coated tissue culture plastic for adhesion‐dependent activation were prepared as mentioned in Gambardella et al.^18^

### 2.4 Analysis of signaling events

Phosphorylation of Akt/PKB (Thr 308) and Erk (Thr 202, Tyr 204) was analyzed by Western blotting with phosphospecific antibodies (Cell Signaling Technology, Leiden, The Netherlands) as described.^19^

### 2.5 Analysis of cell surface receptors

Surface integrins and FcγRs were determined by flow cytometry with specific antibodies for mouse integrins or FcγRs (see Table 1). Data were collected on a LSR Fortessa (BD Biosciences, Oxford, UK) and analyzed using FlowJo V10 software.

**Table 1:** Integrin and FcγR antibodies used in this study

Clone	Specificity	Isotype	Source
HMb1‐1	Mouse integrin β1	Hamster IgG	Thermofisher, Paisley, UK
M/170	Mouse Mac‐1 (α_M_β_2_)	Rat IgG2b	Thermofisher, Paisley, UK
M17/4	Mouse LFA‐1 (α_L_β_2_)	Rat IgG2a	Thermofisher, Paisley, UK
2C9.G2	Mouse integrin β3	Hamster IgG1	Thermofisher, Paisley, UK
AT130‐5	Mouse FcγRII (CD32)	Mouse IgG1	Biorad, Watford, UK
275003	Mouse FcγRIII (CD16)	Rat IgG2a	R&D Systems, Abingdon, UK
9E9	Mouse FcγRIV (CD16.2)	Hamster IgG1	F. Nimmerjahn, University of Erlangen, Germany

### 2.6 ROS production assays

Total ROS production was measured in real‐time by chemoluminescence on a Cytation plate reader (BioTek Instruments, Swindon, UK) in 96‐well polystyrene plates (Nunc, Paisley, UK) essentially as previously described,^18^ analyzing 5 × 10^5^ neutrophils in the presence of 150 μM luminol and 18.75 U/ml exogenous HRP. In some cases, cells were pre‐incubated with 10 μM diphenyleneiodonium (DPI; Cayman Chemicals, Michigan, USA). Data output was recorded as relative light units per second.

### 2.7 Degranulation

Degranulation of neutrophils that had been plated into appropriately coated wells or that had been stimulated by N‐formyl‐methionyl‐leucyl‐phenylalanine (fMLF) in the presence of cytochalasin B were assayed as in^20^ by in‐gel zymography of neat supernatants and immunoassay of diluted supernatants. Due to a lack of mouse lactoferrin standards, lactoferrin release was expressed in arbitrary units.

### 2.8 Chemotaxis

Neutrophil chemotaxis in a 0–300 nM gradient of fMLF was analyzed using a Dunn chemotaxis chamber as mentioned in Vermeren et al.^20^ Cells were time‐lapse imaged using an inverted (Leica, Newcastle, UK) RMDIB microscope with temperature‐controlled chamber. Images were captured every 30 s using micromanager software and an Orca camera (Hamamatsu, Welwyn Garden City, UK). Paths of individual neutrophils were tracked using the “Manual Tracking” plug‐in into ImageJ, for subsequent analysis of the tracks using the “Chemotaxis” plug‐in (Ibidi). Tracks from time‐lapse movies generated on three separate days were combined for analysis.

## 3 RESULTS AND DISCUSSION

**Figure 1:**
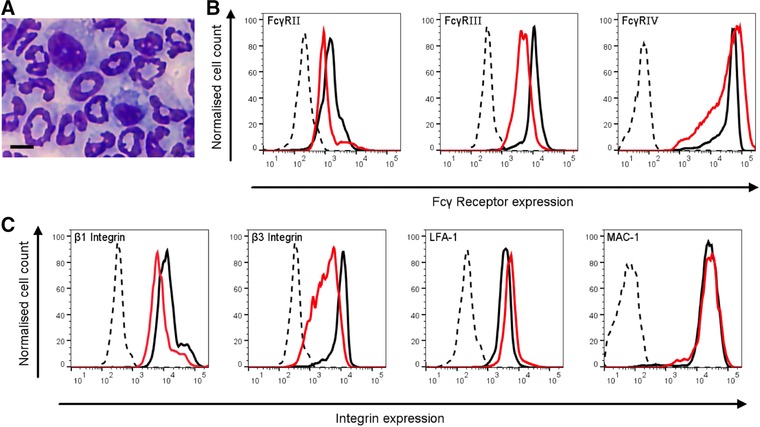
**HoxB8 neutrophils express neutrophil integrins and FcγRs**. (A) A representative cytocentrifuge preparations of Quick‐Diff stained HoxB8 neutrophils after 5 d of differentiation. Scale bar, 5 μm. (B, C) HoxB8 neutrophil surface FcγRs (B) and integrins (C) as indicated were analyzed by flow cytometry. Representative FACS plots are shown from a minimum of 3 separate experiments performed with HoxB8 neutrophils that had been enriched by separation over a discontinuous percoll gradient. Black traces, BMNs; red/grey traces, HoxB8 neutrophils; broken lines, isotype controls; for FcγRIV, broken line represents secondary antibody only control

We aimed to identify a model system that would be amenable to the analysis of molecular events in adhesion‐dependent neutrophil signaling, and replicate neutrophil functions that are controlled by FcγRs and/or integrins. We initially tested DMSO‐induced neutrophil‐like PLB‐985 cells, but had to realize that they are only a poor model for signaling events induced downstream of integrin and/or FcγR ligation (Supplemental Fig. 1). This might be because PLB‐985 cells lack expression of FcγRIII/CD16^21^ a finding that was confirmed by ourselves (Supplemental Fig. 2). We therefore set out to test whether HoxB8 neutrophils might represent a suitable model for neutrophil signaling and functions that depend on integrins and/or FcγRs. We generated HoxB8 neutrophil populations from conditionally immortalized bone marrow‐derived progenitor cells. HoxB8 progenitor populations readily differentiated upon withdrawal of 4OHT, with neutrophils identified by their characteristic nuclear morphology in cytocentrifuge preparations (Fig. 1A).

### 3.1 HoxB8 neutrophils express neutrophil integrins and FcγRs

Mouse neutrophils constitutively express two activating FcγRs, FcγRIII, and FcγRIV, as well as the inhibitory FcγRIIB. We compared FcγR expression by BMNs and differentiated HoxB8 neutrophils (Fig. 1B), noting both expressed all three FcγRs. In addition to the major leukocyte β2 integrins LFA1/αLβ2 and Mac1/αMβ2, neutrophils express a range of leukocyte‐specific and ubiquitous integrins. We compared the surface expression of the abundant leukocyte integrins LFA1 and Mac1 as well as β1 and β3 subunits on BMNs and HoxB8 neutrophils. Both expressed all of these integrins at comparable levels (Fig. 1C).

### 3.2 Stimulating HoxB8 neutrophil integrins and FcγRs induces cellular signaling

**Figure 2:**
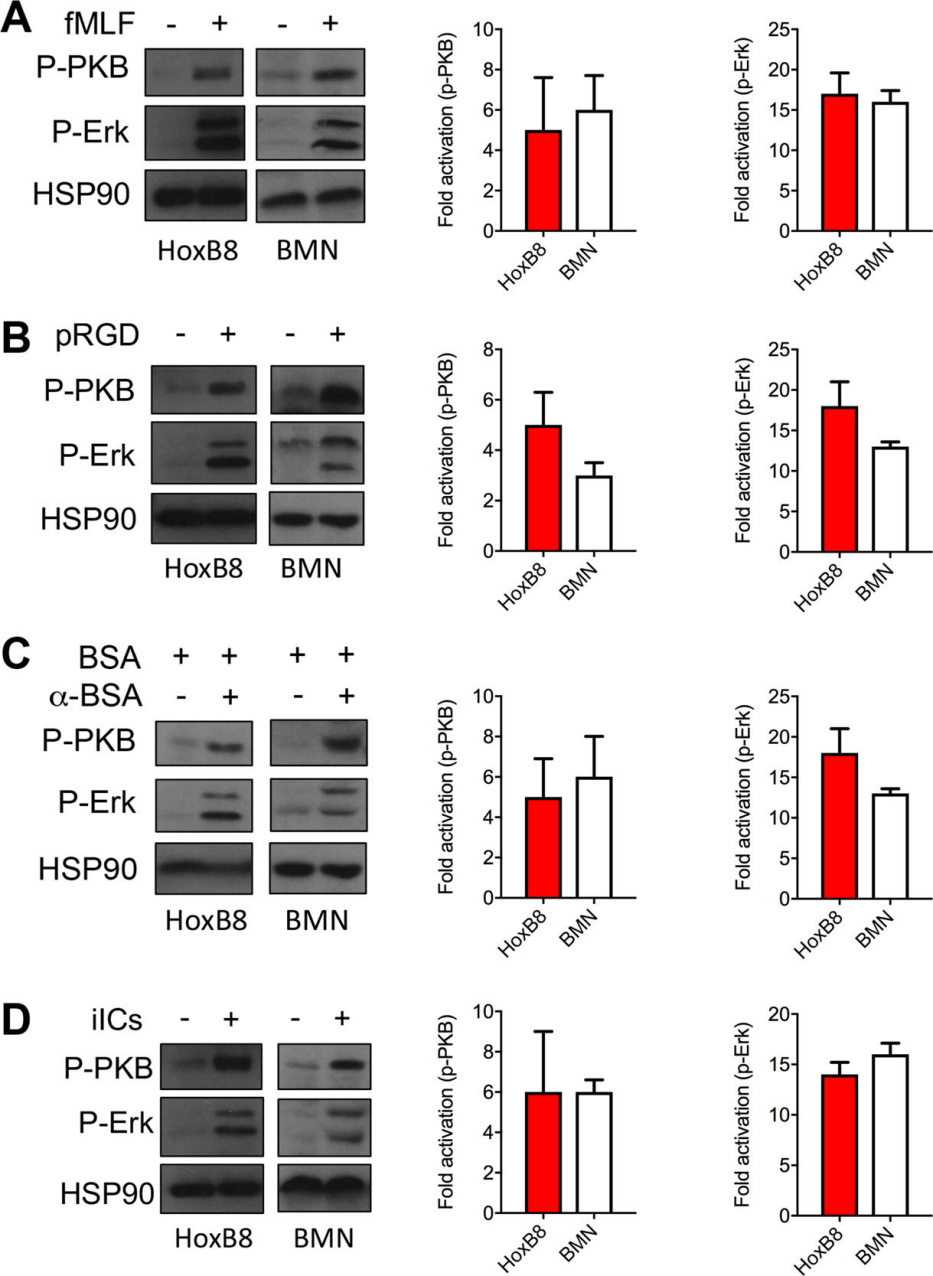
**Integrin and FcγR stimulation‐induced intracellular signaling events in BMNs and HoxB8 neutrophils**. BMNs and HoxB8 neutrophils (from 3 different bone marrow donors) were stimulated (A) for 1 min with 1 μM fMLF in suspension, or by being plated onto (B) pRGD or (C) immobilized ICs for 12 min, or (D) for 10 min with 10 μg/ml insoluble ICs in suspension. Signaling events were analyzed by Western blotting. Hsp90 served as a loading control. Representative blots are shown. Images were put into greyscale for ease of viewing after densitometry. Densitometric analysis was carried out to calculate the fold activations obtained in individual experiments. Mean fold activations (±sem) obtained with HoxB8 neutrophils and BMNs under all the stimulation conditions of at least 4 separate experiments are plotted. Statistical analysis was by unpaired *t* test

FcγRs and integrins utilize convergent signaling pathways in neutrophils.^3^, ^22^ We were keen to identify a cellular model that replicates signaling events that occur upon integrin and/or FcγR stimulation in primary neutrophils. As a control, we analyzed fMLF‐stimulated intracellular signaling events indirectly by Western blotting of cell lysates with phosphospecific antibodies specific for the activated forms of PKB/Akt (a read‐out for phosphoinositide 3‐kinase) and Erk. fMLF stimulation resulted in rapid phosphorylation of Akt/PKB and Erk in BMNs and HoxB8 neutrophils (Fig. 2A). To analyze integrin‐dependent stimulation, we next plated the cells onto the synthetic integrin ligand polyArg‐Gly‐Asp (pRGD), which mediates robust neutrophil stimulation even in the absence of costimulation of another receptor.^23^ This triggered phosphorylation of Akt/PKB and Erk in HoxB8 neutrophils to a comparable extent as was observed with freshly prepared BMNs (Fig. 2B). We also stimulated the cells by plating them onto immobilized ICs, again obtaining comparable fold activations of Akt/PKB and Erk with HoxB8 neutrophils and with BMNs (Fig. 2C). Finally, we also stimulated the cells with insoluble ICs. This again resulted in Akt/PKB phosphorylation and Erk phosphorylation with HoxB8 cells and with freshly prepared BMNs (Fig. 2D). These results, in combination with the poorer responses obtained with PLB‐985 (Supplemental Fig. 1) led us to conclude that HoxB8 neutrophils do indeed represent a suitable model system for the analysis of FcγR/integrin‐induced neutrophil signaling. We next tested whether HoxB8 cells are able to perform neutrophil functions that require integrins or that are induced downstream of integrin and/or FcγR ligation.

### 3.3 HoxB8 neutrophils generate ROS in response to integrin and/or FcγR stimulation

**Figure 3:**
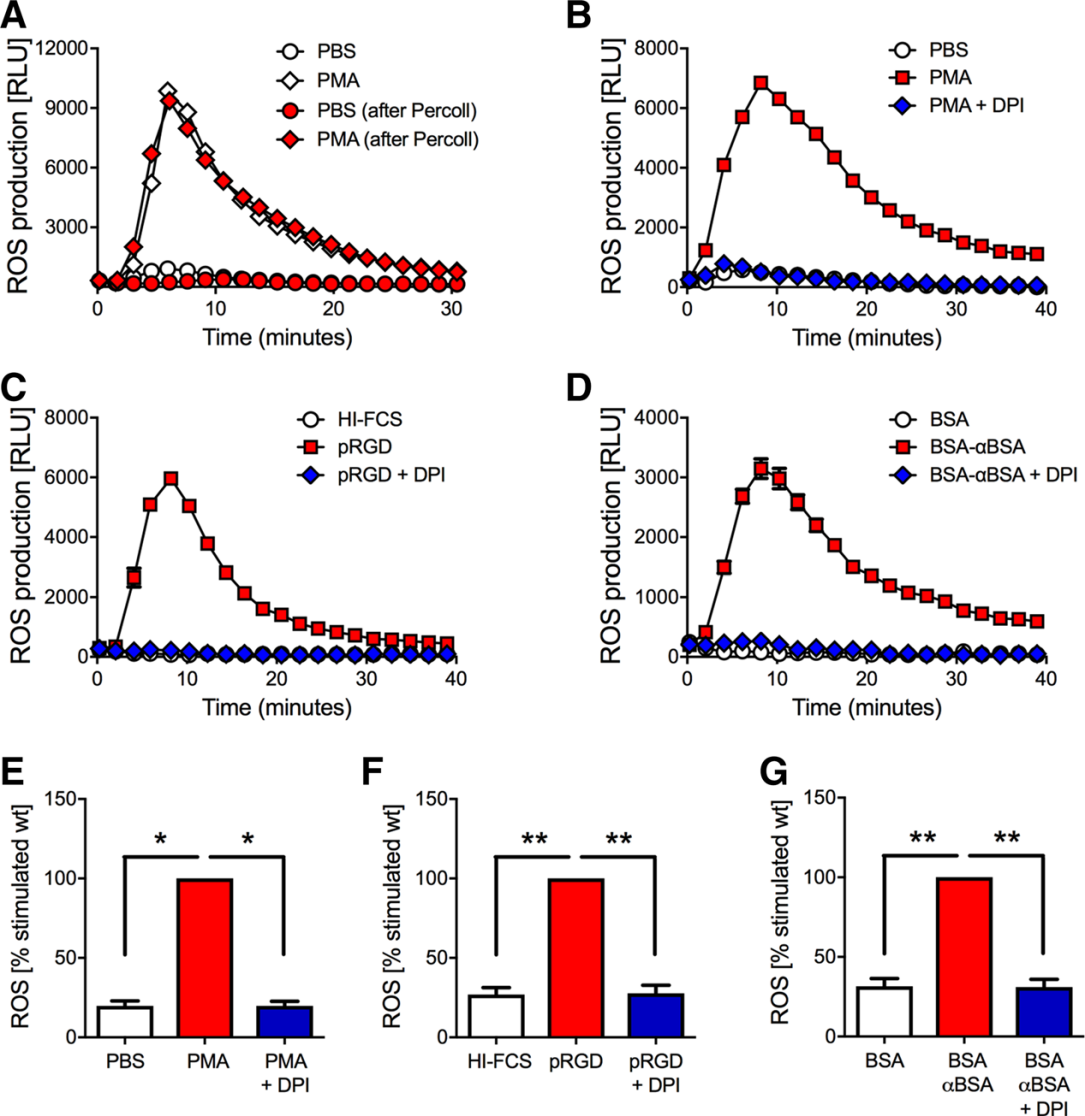
**HoxB8 neutrophils generate ROS upon integrin/FcγR stimulation**. (A) HoxB8 neutrophils were or were not subjected to purification by percoll gradient before being stimulated with 100 nM PMA or its vehicle for ROS assays. A representative example is shown. Red symbols, no percoll gradient; blue symbols, with percoll gradient (B–G). ROS generation of percoll‐enriched HoxB8 cells that had or had not been pre‐incubated with DPI prior to stimulation with PMA (B, E), being plated onto pRGD (C, F), or immobilized ICs (D, G). Representative examples (mean ± range) from a minimum of 4 separate experiments are plotted (B–D). Integrated results are plotted normalized to the activated condition (E–G). *P < 0.05; **P < 0.01; statistical analysis was by *t* test. Results shown in this figure were obtained with HoxB8 neutrophils from a single donor, and reflect those obtained with cells obtained from two other donors on a different plate reader

HoxB8 neutrophils have already been shown to produce ROS when stimulated with PMA^12^ or with serum‐opsonized zymosan.^13^ We confirmed that PMA stimulation of HoxB8 neutrophils resulted in significant ROS production as determined in a luminol‐amplified chemoluminescence assay, which has been shown to detect mostly the production of superoxide anions.^24^ Using this sensitive functional assay, we were able to show that the same number of HoxB8 cells produced the same ROS in response to PMA stimulation (Fig. 3A), regardless of whether they had or had not been subjected to neutrophil enrichment by discontinuous percoll gradient. PMA‐induced ROS production by HoxB8 neutrophils was abrogated by pretreatment of HoxB8 neutrophils with the NADPH oxidase inhibitor DPI (Fig. 3B, E). We also tested ROS production in response to integrin and/or FcγR ligation, and found HoxB8 neutrophils also produce significant, NADPH oxidase‐dependent ROS upon being plated onto immobilized ICs or onto the synthetic integrin ligand pRGD (Fig. 3C, D, F, G).

### 3.4 HoxB8 neutrophils degranulate in response to integrin and/or FcγR stimulation

**Figure 4:**
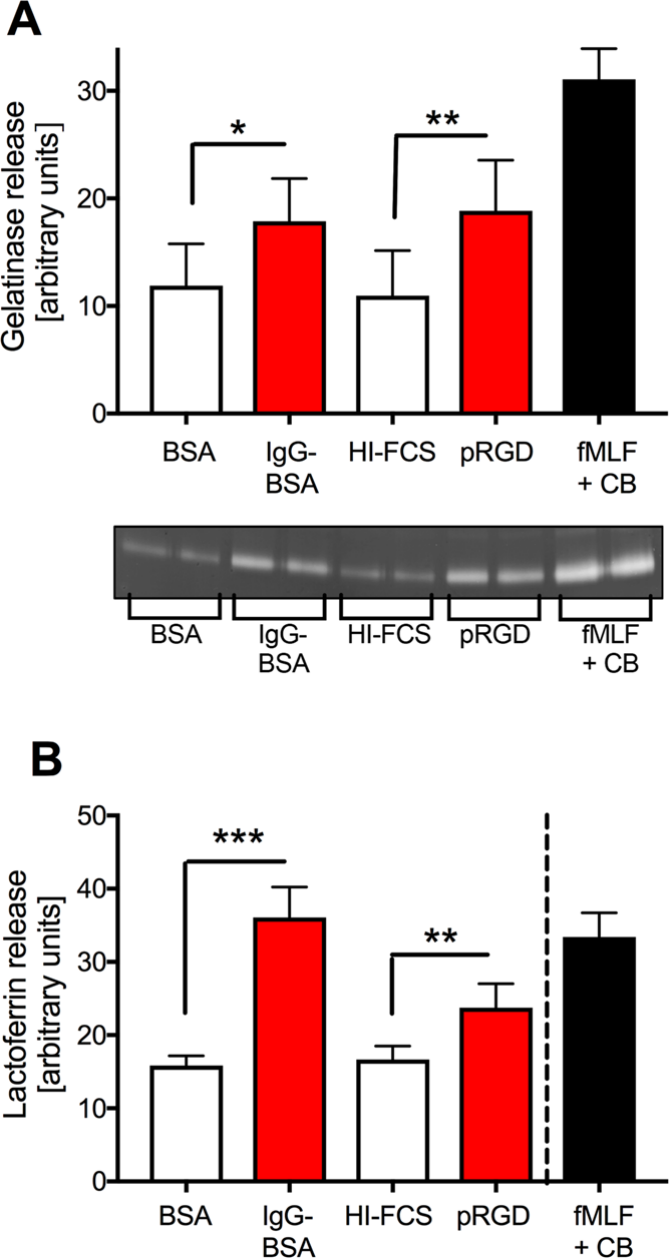
**HoxB8 neutrophils degranulate upon integrin/FcγR stimulation**. HoxB8 neutrophils were stimulated by being plated onto pRGD or immobilized ICs, or with 1 μM fMLF in the presence of 10 μM cytochalasin B for 1 h. Released gelatinase (A) and lactoferrin (B) in the supernatant was determined by in gel‐zymography (A) and ELISA (B). A broken line in (B) indicates that the readings obtained with fMLF and cytochalasin B were obtained with more dilute supernatants. Bars show mean ± sem from at least 3 separate experiments performed with HoxB8 neutrophils obtained from 2 different bone marrow donors. *P < 0.05; **P < 0.01; ***P < 0.001; statistical analysis was by paired *t* test (A) and by unpaired *t* test (B)

Degranulation, in particular of secondary granules, is poor in neutrophil‐like cells (e.g. HL60 and PLB‐985).^21^, ^25^ To our knowledge, degranulation had not yet been tested with HoxB8 neutrophils. We therefore stimulated HoxB8 neutrophils by plating them onto pRGD or immobilized ICs to test whether this caused HoxB8 neutrophils to release gelatinase from tertiary granules. As a control, we also stimulated the cells with fMLF in the presence of cytochalasin B, which drives significant degranulation to the outside of the cell.^26^ This treatment triggered strong gelatinase granule release in HoxB8 neutrophils, with weaker gelatinase release from HoxB8 neutrophils observed with the more physiologic stimuli (Fig. 4A). We also analyzed degranulation from secondary granules by determining lactoferrin release. Lactoferrin release was also induced by plating of the HoxB8 neutrophils onto pRGD or immobilized ICs, or, under the control conditions, fMLF in the presence of cytochalasin B (Fig. 4B). We note, that lactoferrin, but not gelatinase release was induced more potently by immobilized ICs than by pRGD. In summary, we have provided two examples of functional responses, ROS generation and degranulation from secondary and tertiary granules, that are triggered upon adhesion‐dependent stimulation of primary neutrophils and that are emulated by HoxB8 neutrophils.

### 3.5 HoxB8 neutrophil chemotaxis

**Figure 5:**
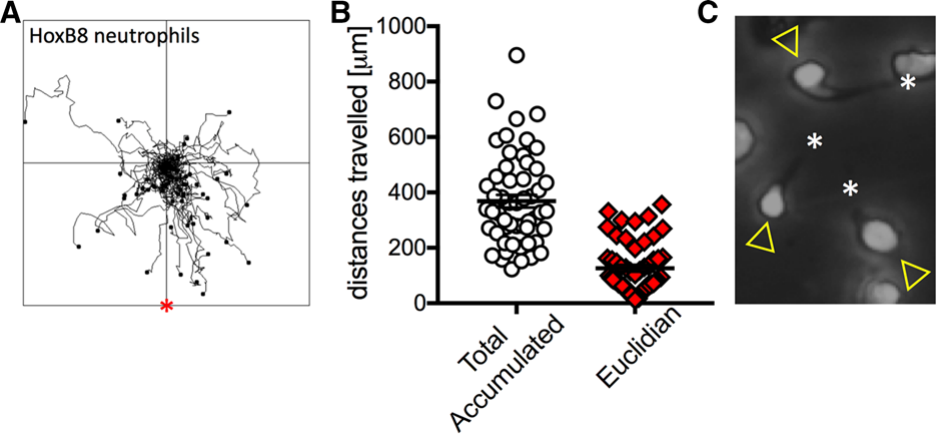
**HoxB8 neutrophil chemotaxis**. HoxB8 neutrophils were allowed to chemotax toward fMLF. (A) Tracks of individual cells from experiments performed on three different days are combined in this spider plot. * indicates the source of chemoattractant. (B) Total and Euclidian distances travelled by the cells shown in (A) are plotted. Error bars show sem. (C) Cropped still from a time‐lapse movie showing 3 HoxB8 neutrophils with characteristic migrating morphology comprising leading edge (arrowhead) and trailing end (asterisk)

Reaching inflammatory sites by chemotaxis, neutrophils are among the fastest moving single cells in the body. All forms of cell migration involve the integrin dependent, repeated attachment and detachment of the cell to and from the substratum. In addition, neutrophils need to recognize the chemotattractant gradient, polarize and migrate persistently toward the source of the gradient. The degree of integrin dependency of chemotaxis depends upon the experimental system used to analyze chemotaxis. HoxB8 neutrophils have already been shown to be able to chemotax in transwells,^13^ which are thought to support integrin‐independent chemotaxis.^27^ We performed HoxB8 neutrophil chemotaxis in Dunn chambers, where cells migrate between sheets of glass in a gradient of chemoattractant in an integrin‐dependent fashion. Our experiments showed directional migration of HoxB8 neutrophils toward fMLF (Fig. 5A, B). The total accumulated and Euclidian distances travelled by HoxB8 neutrophils in these experiments were in keeping with those of BMNs in Dunn chambers analyzed under the same conditions.^18^ During migration, chemotaxing HoxB8 neutrophils displayed the typical polarized morphology that is characterized by a distinct leading edge and a trailing end (Fig. 5C). This suggests that HoxB8 cells represent a suitable model system for the analysis of neutrophil chemotaxis.

In summary, we have shown here that HoxB8 neutrophils, unlike neutrophil‐like differentiated PLB‐985 cells, were able not only to replicate FcγR and/or integrin‐dependent signaling events, but also neutrophil functions, including degranulation from secondary and tertiary granules, triggered downstream of integrin and/or FcγR ligation.

HoxB8‐immortalized hematopoietic progenitors are easy to generate from mouse bone marrow stem cells; conditionally immortalized progenitors can be kept in culture for months. HoxB8 progenitors can be generated from existing mouse mutants, including those that are embryonic lethal, with reports that fetal liver and ES cells can also be used to generate the progenitors,^28^ thus providing the investigator with a lot of flexibility. HoxB8 progenitors have already been shown to be amenable to lentiviral/retroviral transduction, including shRNA‐mediated gene knockdown, clustered regularly interspaced short palindromic repeats (CRISPR)‐mediated knockout as well as heterologous expression of proteins of interest.^11^, ^13^, ^14^ Lentiviral modification of HoxB8 progenitors can be achieved in a fraction of the time, and at much lower cost than the generation of a new mouse line from which to prepare primary neutrophils. With published evidence suggesting that HoxB8 progenitors both before and after differentiation are also suitable for adoptive transfer,^13^, ^29^ the potential uses of this approach even include the analysis of neutrophil recruitment in vivo. HoxB8 neutrophils therefore represent an attractive option for the analysis of aspects of neutrophil biology without the dependence on large numbers of laboratory mice, nor the need of generating new lines in order to analyze new genetic modifications.

## Supporting information

Supporting Information

Supporting Information

Supporting Information
