# Supplementary material for: HoxB8 neutrophils replicate Fcγ receptor and integrin‐induced neutrophil signaling and functions
Source: J Leukoc Biol. 2018 Sep 13;105(1):93–100. doi: 10.1002/JLB.1AB0618-232R (PMC6348421; doi:10.1002/JLB.1AB0618-232R)
Supplement: Supplementary file 1 — Supporting Information [file JLB-105-93-s001.docx]

**J. Chu et al., HoxB8 neutrophils replicate Fcγ receptor and integrin-dependent neutrophil signalling and functions; Supplemental Material**

**Supplemental Methods**

***HoxB8 neutrophils***

Cells were cultured at 37ºC with 5% CO_2_ in a humidified incubator. A retroviral transfer vector encoding ER-HoxB8 was provided by Hans Häcker (St Jude’s Children’s Research Hospital, Memphis). Retroviruses were generated by calcium phosphate transfection (BD CalPhos kit) of the ecotrophic Plat-E packaging cells [1] as per manufacturer’s instruction. HoxB8 conditionally immortalised progenitors were generated from bone marrows of C57Bl/6 mice essentially as described [2]. Progenitor cells were enriched using a Lin- selection kit (Miltenyi Biotech), pre-stimulated with 25 ng/ml SCF, 10 ng/ml IL-3 and 20 ng/ml IL-6 (all Biolegend) in IMDM with 15% heat-inactivated FBS, 1% penicillin/streptomycin, 1% glutamine for 48 hours. Progenitors were transduced by spinoculation with ER-Hoxb8 retrovirus in 12.5μg/ml retronectin (Takara) coated non-tissue culture-treated dishes (Nunc) with 5μg/ml polybrene (1000*g*, 30 minutes, 25ºC) in growth medium (RPMI-1640 with 10% FBS, 1% penicillin/streptomycin, 1% glutamine, 10 ng/ml GM-CSF (BioLegend) and 200nM 4-hydroxy-tamoxifen (4OHT). Transduced progenitors were selected with 0.6mg/ml G418 for 5 days. For differentiation, conditionally immortalised progenitors were washed extensively in PBS before being resuspended in growth medium lacking 4OHT for 5 days. GM-CSF differentiation of the progenitors resulted in mixed populations of neutrophils (~60%) and monocytes/macrophages. Increased neutrophil purity (>80%) could be obtained by enriching the differentiated neutrophils by discontinuous percoll gradient (25%, 42% and 51% [3]) with HoxB8 neutrophils separating at the 25% / 42% percoll interface. We also immortalised HoxB8 progenitors in OptiMEM supplemented with 10% FBS, 1% penicillin/streptomycin, 1% glutamine, 30μM β-mercaptoethanol, 10 ng/ml SCF (Peprotech) and 4OHT, differentiating these progenitors OptiMEM growth medium lacking 4OHT that was supplemented with 10-50 ng/ml SCF alone or with 20ng/ml SCF and G-CSF (Peprotech) each. In our hands, SCF-supported HoxB8 progenitors (and HoxB8 neutrophils) grew significantly less robustly than GM-CSF ones. Prior to experiments cells were washed into assay buffer.

***PLB-985 cell culture.***

PLB-985 cells were cultured in RPMI-1640 supplemented with 10% FBS, 1% penicillin/streptomycin and 1% glutamine. PLB-985 cell differentiation was induced by incubation for 5 days in growth medium supplemented with 1.25% DMSO.

***Isolation of primary human neutrophils.***

Human neutrophils (PMNs) were isolated from healthy donor blood by dextran sedimentation followed by discontinuous percoll gradient (25%, 42% and 51%) as previously described [4]. Human neutrophil purity was >95% according to cytocentrifuge preparations. Ethics approval for work with human blood was obtained from the local Lothian Research Ethics Committee (approval AMREC 15-HV-013).

**Table S1. *Integrin and FcγR antibodies used in this study***

| **Clone** | **Specificity** | **Isotype** | **Source** |
| --- | --- | --- | --- |
| LM2 | Human Mac-1 (α_M_β_2_/CD11b) | Mouse IgG1 | Ian Dransfield |
| TS1/18 | Human integrin β_2_ /CD18 | Mouse IgG1 | Ian Dransfield |
| WAC70 | Human LFA-1 (α_L_β_2_ **/**CD11a) | Mouse IgG2A | Ian Dransfield |
| AK7 | Human integrin α2 | Mouse IgG1 | Serotec (now Biorad) |
| DJ130c | Human FcγRIIIb (CD16) | Mouse IgG1 | Dako (now Agilent) |
| 8.26 | Human FcγRIIa (CD32) | Mouse IgG2b | BD BioSciences |
| 10.1 | Human FcγRI (CD64) | Mouse IgG1 | Dako (now Agilent) |

**Supplemental Figure Legends.**

**Figure S1. Differentiated PLB-985 are a poor model for integrin / FcγR-dependent neutrophil signalling.** Freshly prepared human neutrophils (PMNs) and DMSO-induced PLB-985 cells were stimulated (A) with 100nM fMLF, by being plated onto (B) the β2 integrin ligand fibrinogen with co-stimulation by TNFα, (C) onto immobilised ICs, or (D) with 10 μg/ml insoluble ICs. Lysates were prepared as detailed in Materials and Methods. Clarified lysates were subjected to SDS-PAGE and Western blotting for analysis of cellular signalling events with phosphospecific antibodies. Western blots shown are representative examples of a minimum of 3 separately conducted experiments. Densitometric analysis of all individual blots was carried out for calculation of fold activations in the individual experiments. The fold activations (±SEM) obtained with differentiated PLB-985 and PMNs are plotted. PMNs reached higher fold activations throughout; where indicated this reached statistical significance (analysis by unpaired t-test; *, p<0.05).

**Figure S2.** **PLB-985 cells lack expression of CD16.** DMSO-differentiated PLB-985 cells and freshly prepared healthy donors blood derived PMNs were labelled for cell surface FcγRs (A) and integrins (B; see table S2 for antibodies) for analysis by flow cytometry. Representative FACS plots are shown from a minimum of three separate experiments performed. Black traces, PMNs; red traces, PLB-985s; broken lines, isotype controls (for FcγRs, where antibodies where directly coupled) or secondary antibody only controls (for integrins, where uncoupled primary antibodies were used).

***Supplemental References***

1. Morita, S., Kojima, T., Kitamura, T. (2000) Plat-E: an efficient and stable system for transient packaging of retroviruses. Gene Ther 7, 1063-6.

2. Wang, G. G., Calvo, K. R., Pasillas, M. P., Sykes, D. B., Hacker, H., Kamps, M. P. (2006) Quantitative production of macrophages or neutrophils ex vivo using conditional Hoxb8. Nat Methods 3, 287-93.

3. Haslett, C., Guthrie, L. A., Kopaniak, M. M., Johnston, R. B., Jr., Henson, P. M. (1985) Modulation of multiple neutrophil functions by preparative methods or trace concentrations of bacterial lipopolysaccharide. Am J Pathol 119, 101-10.

4. Chu, J. Y., Dransfield, I., Rossi, A. G., Vermeren, S. (2016) Non-canonical PI3K-Cdc42-Pak-Mek-Erk Signaling Promotes Immune-Complex-Induced Apoptosis in Human Neutrophils. Cell Rep 17, 374-386.
