# Supplementary figures and images for: HoxB8 neutrophils replicate Fcγ receptor and integrin‐induced neutrophil signaling and functions
Source: J Leukoc Biol. 2018 Sep 13;105(1):93–100. doi: 10.1002/JLB.1AB0618-232R (PMC6348421; doi:10.1002/JLB.1AB0618-232R)

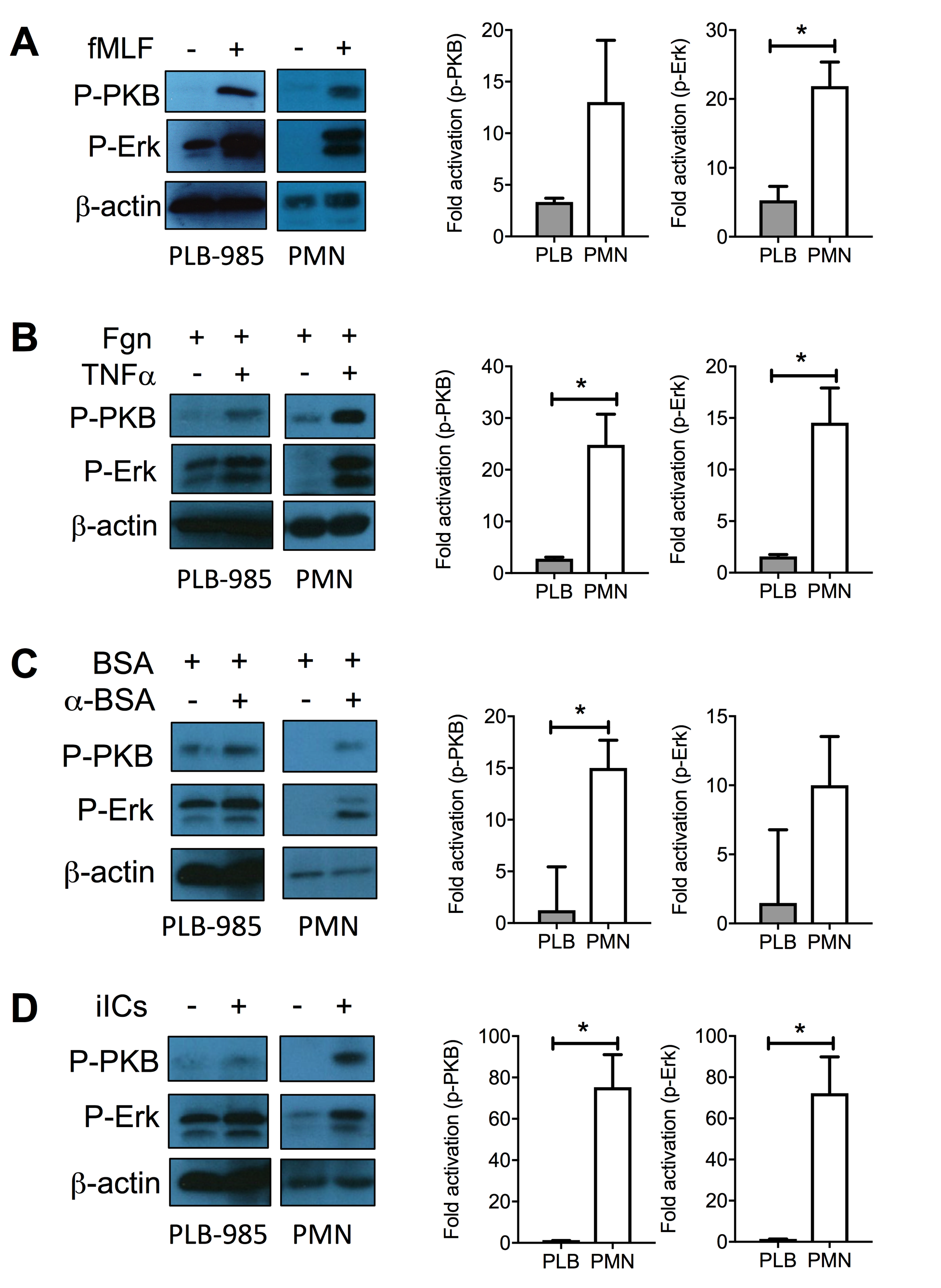

Supplement: Supplementary file 2 — Supporting Information [file JLB-105-93-s002.tiff]

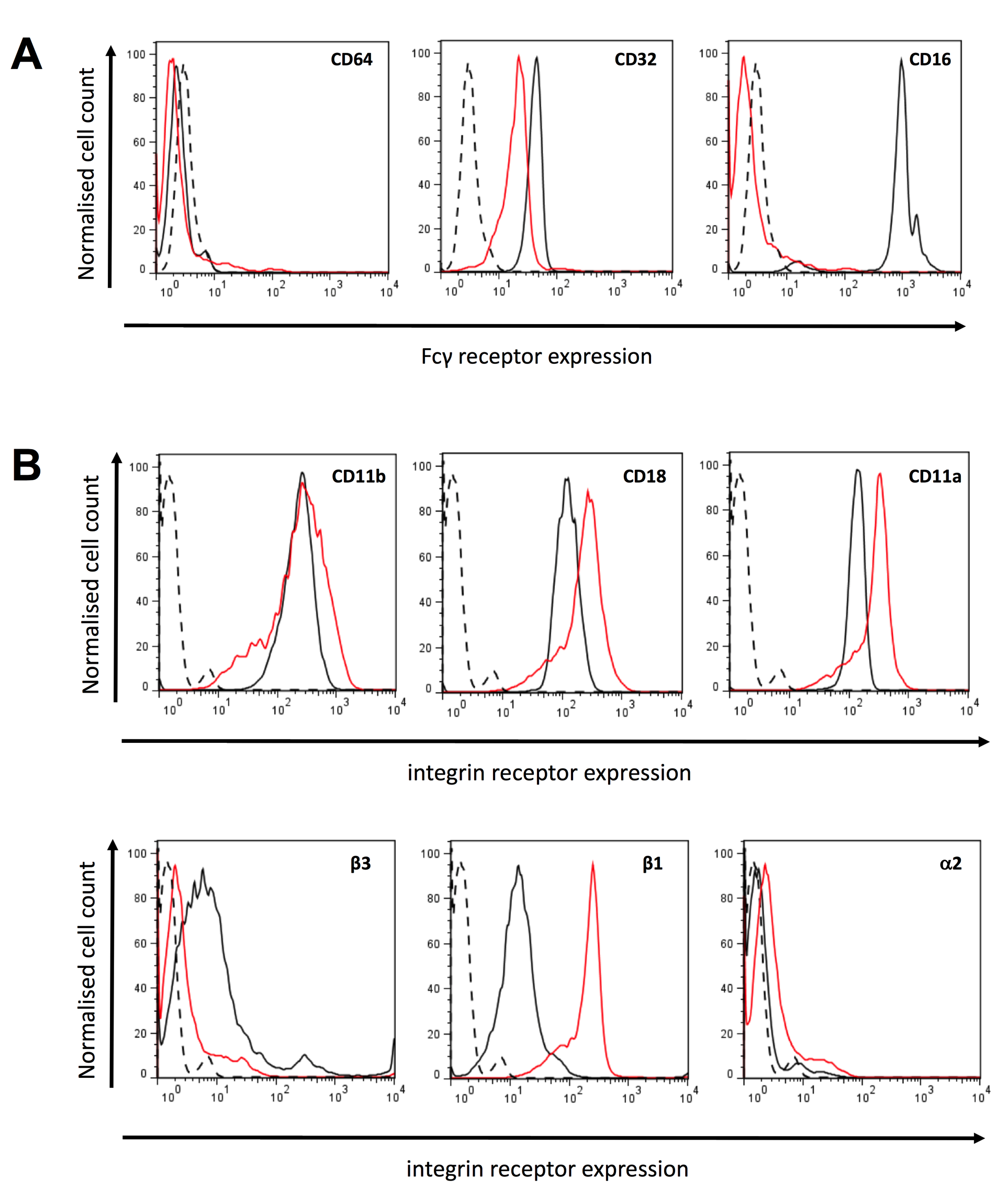

Supplement: Supplementary file 3 — Supporting Information [file JLB-105-93-s003.tiff]
